# Religious, socio-cultural norms and gender stereotypes influence uptake and utilization of maternal health services among the Digo community in Kwale, Kenya: a qualitative study

**DOI:** 10.1186/s12978-020-00919-6

**Published:** 2020-05-24

**Authors:** Vernon Mochache, George Wanje, Lucy Nyagah, Amyn Lakhani, Hajara El-Busaidy, Marleen Temmerman, Peter Gichangi

**Affiliations:** 1 International Centre for Reproductive Health, P.O. Box 91109-80103, Mombasa, Kenya; 2 University of Ghent, Ghent, Belgium; 3 Department of Medical Microbiology, University of Nairobi, Mombasa Field Site, P.O Box 91276-80103, Mombasa, Kenya; 4 Community Health Department, Aga Khan University, P.O Box 83013-80100, Mombasa, Kenya; 5 Department of Health, County Government of Kwale, P.O Box 6-80403, Kwale, Kenya; 6 Aga Khan University Hospital, 3rd Parklands Avenue, Limuru Road, Nairobi, Kenya; 7 Technical University of Mombasa, P.O Box 90420-80100, Mombasa, Kenya

**Keywords:** Demand-side factors, Religious and socio-cultural norms, Gender stereotypes, Maternal health, Digo, Kwale

## Abstract

**Background:**

Maternal health outcomes in resource-limited settings are typically influenced by supply-side factors affecting the provision of quality health services. The extent to which demand-side factors contribute to this influence is unclear. We aimed to explore how individual and community-wide factors influenced uptake and utilization of maternal health services among the Digo community residing in Kwale County of coastal Kenya.

**Methods:**

Between March and December 2015, we conducted 5 focus group discussions (FGDs) and 15 in-depth interviews (IDIs) with members of the Digo community predominant in Kwale county, Kenya. Respondents were sampled purposively and included female (pregnant and postpartum) as well as male adult community members. A thematic content analytic approach was used.

**Results:**

There were a total of 47 FGD respondents, including 15 (32%) females with a median (interquartile, IQR) age of 38 (27–55) years and 6 (3–8) children. Majority (40%) reported attaining secondary-level education. All IDI respondents were female with a median (IQR) age of 27 (24–35) years and 4 (2–5) children. Majority (80%) had attained primary-level education. We found that religious and socio-cultural norms as well as gender stereotypes were important influences on the uptake and utilization of maternal health services, including facility-based delivery and contraception. Key amongst this was the unspoken deference to the counsel of a prominent matriarchal figure in the decision-making process.

**Conclusions:**

Among the Digo community of coastal Kenya, a unique social-cultural context comprising of a religious and gendered value belief system influences women’s reproductive health and rights. These findings highlight the important role of demand-side factors in influencing maternal health outcomes. In addition to addressing supply-side factors, programs in such settings should aim to address factors that leverage inherent social capital to drive demand for maternal health services ensuring that they are not only effective, but also responsive to the local context.

## Plain ENGLISH summary

Many programs looking to improve the health of mothers often aim to address how health services are delivered e.g. training and employing more health providers. Most of these programs rarely address factors that affect whether or not communities will take up or use these services and why. We aimed to address this by conducting focus group discussions with community members as well as in-depth interviews with pregnant and postpartum mothers among the Digo, who are the predominant community living in Kwale county of coastal Kenya so as to understand factors at individual and community level that could influence whether community members take up or use these services. We found that in this community, specific standards related to their religion and culture as well as widely-held beliefs about the role of men versus women, were an important influence on the uptake and utilization of these services. Specifically, we found that a prominent mother-figure in the community was key in determining whether or not women delivered in a health facility or used contraception. Our findings highlight the importance of implementing programs that address and respond to unique socio-cultural contexts within similar settings if they are to be effective and sustainable.

## Background

Socio-cultural norms and gender stereotypes have been known to influence the uptake and utilization of maternal health services [1–3]. This is thought to be through the effect of gendered social roles, the dynamics of vulnerability inherent in existing social structures, as well as negative socio-cultural norms [4, 5]. The resultant outcome is attitudes and behaviors that affect, among others, pregnancy intention and attendant uptake of contraceptive services; as well as perceptions of what constitutes a normal birthing process, and the eventual decision-making regarding choice of place of delivery [6].

Gendered social roles affect the extent to which men get involved in sexual and reproductive health-related matters [7, 8]. Gender dynamics also determine the level of autonomy that women have over decisions regarding their health [9]. In majority of communities in sub-Saharan Africa, which are predominantly patriarchal, men are the principal decision-makers, directly or indirectly influencing women’s access to and utilization of health services [10, 11]. In contrast, women have to bear with societal attitudes and expectations that confer an undue health disadvantage.

Social vulnerability influences the ability of certain population segments to recover from the adverse impacts of different stressors and has been shown to have inter-generational consequences [12–14]. In this regard, poor educational, health and developmental outcomes have been described in societies where negative socio-cultural norms relegate women to second class members of the community [15–17]. Conversely, better health outcomes have been associated with taking into consideration the gender implications of any policies, actions and programs while incorporating cultural and gender-appropriate interventions [18, 19].

In coastal Kenya, the effects of this socio-cultural and gender influence are apparent. According to the Kenya Demographic and Health Survey (KDHS) 2014, Kenya’s Coast Province reported the second lowest modern contraceptive prevalence rate in the country at 38.3% with more than half of respondents not using any contraceptive method [20, 21]. Additionally, more than a third (34.1%) of male respondents believed a woman using contraception will become promiscuous. Nearly half (41.1%) of pregnant women in this setting reported having given birth at home.

Further, more than half (50.5%) of women in this setting did not get a postnatal check-up as recommended within the first two days of delivery [22]. This is probably related to local norms requiring a woman to spend the first forty days after delivery in isolation so as to ward off potential ‘evil eyes’. It is instructive that while nearly all male respondents (97.2%) were responsible for making decisions regarding their health, just about three-quarters (76%) of women reported being able to do this.

Among women who delivered at home, Coast Province had the highest proportion (36.1%) reporting that they did not think it was important to deliver in a health facility. In line with this, the province also had the lowest proportion of women who reported that they delivered at home because the health facility was either too far or they lacked transport (32.5%). An even smaller proportion (14.3%) reported that their home delivery was influenced by financial considerations.

This study was conducted within the context of implementing two large-scale, maternal health programs funded by the European Commission in Kwale County. The Missed Opportunities in Maternal and Infant health project was a five-year operations research study with the overall objective of improving maternal and newborn health by utilizing missed opportunities in the postpartum period through a combined facility and community-based strategy [23]. The Mama Na Mtoto II project was the second phase of a four-year, maternal and child health initiative which aimed to contribute towards a reduction in maternal and childhood deaths through strengthening community health systems [24].

Given the opportunity afforded by these programs, we sought to understand the individual and community-wide factors that could influence uptake and utilization of maternal health services in this setting. Specifically, we aimed to explore if and how religious and socio-cultural norms as well as gender stereotypes could influence uptake and utilization of maternal health health services. Given that the scope of maternal health care is broad encompassing contraception, pre-conception, antenatal, delivery and postnatal care, our enquiry specifically focused on the uptake of contraception, antenatal and postnatal services, as well as choice of place of delivery. This was mainly informed by the focus of the initial programs and also the regularly reported poor outcomes in this setting for these services.

## Methods

### Study setting and population

This study was conducted between March and December 2015 in Kwale County, located on the southern coastal strip of Kenya between Mombasa County and the border with Tanzania. According to the 2009 Kenya national census, Kwale had a population of ~ 650,000 with a preponderance of females [25]. Majority of the population is rural-based with very poverty levels upwards of 70%. The predominant community in our study setting is the Digo; one of the nine sub-tribes of the Mijikenda ethnic group in coastal Kenya and northern Tanzania. The main sources of livelihood include fishing, farming, trading and tourism. Kwale County comprises of four sub-counties namely, Matuga, Msambweni, Kinango and Lungalunga.

Data collection focused in two sub-counties (Msambweni and Matuga) where the Digo community predominantly resides. The main language spoken by this community is *Chidigo*, a Bantu derivative, and the predominant religion (80%) is Islam. We have previously shown in this setting that higher educational levels and pregnancy intention were associated with better uptake of contraception and facility-based delivery [26, 27]. Our previous research work in this setting has also demonstrated that active community involvement in maternal health programs significantly improved uptake and utilization of these specific services [28].

### Sampling and recruitment

Study participants were recruited purposively, a strategy that has been used widely in qualitative studies and aims to get a deeper understanding of phenomena by ensuring that a diverse array of perspectives on the topic is included in the sample until attainment of thematic saturation [29, 30]. Specifically, data collection involved focus group discussions (FGDs), as well as in-depth interviews (IDIs). FGD participants were recruited through the efforts of community health volunteers (CHVs) who had been trained as research assistants. To ensure there was no selection bias by the CHVs, every other woman or man was approached for study participation. This was done during communal meetings, including chief’s *barazas* and community dialogues days. Community members who act as ‘gatekeepers’ i.e. chiefs, village elders and local religious leaders, were also invited to participate in the FGDs.

Participants were recruited from different villages in sub-locations within the two sub-counties including Majoreni and Bumbani in Pongwe/Kikoneni ward; Mazumalume in Tsimba Golini ward; Matuga, Ng’ombeni and Kombani in Waa ward; Simkumbe and Mkoyo in Tiwi ward; Kwale town in Kubo South ward as well as Mkomba, Kizibe and Mtsamviani in Mkongani ward so as to get a varied response from the wider Digo community. This varied geographical sampling was aimed at developing a nuanced understanding of the local socio-cultural, religious and gender norms that might influence utilization of maternal health services.

Pregnant and postpartum women were approached at the local health facility by CHVs as they sought care and requested to participate in the IDIs. We envisioned that IDIs among pregnant and postpartum mothers would provide first-hand experiences regarding their decision-making process for seeking care of previous and current pregnancies as well as the delivery and contraceptive options available to them. All participants provided informed consent prior to any study procedures and were given an opportunity to refuse participation without consequence.

### Data collection

As part of a wider study, the principal investigator (VM) spent a significant amount of time in the community observing and documenting various aspects of the Digo community’s social structure and cultural norms. Interviews were primarily conducted by the principal investigator and a research assistant familiar with the local *Chidigo* and Swahili languages and trained in qualitative research methods acted as the note-taker. Interviews took place in a private area in the local shopping center/market or at the local health facility. The FGDs were stratified by age and gender to ensure cultural-appropriateness and promote effective discussions. All interviews were guided by use of semi-structured topic guides which allowed probing and exploration of the different topics under consideration. Each IDI lasted between 45 and 60 min, whereas each FGD took approximately one and a half hours. All interviews were audio-recorded. There was no refusal from potential participants to take part in either IDIs or FGDs.

### Data analysis

Audio-recordings of the IDIs and FGDs were transcribed and translated into English in readiness for analysis. The transcripts were reviewed by the principal investigator to ensure content was accurately represented. VM and GW reviewed the transcripts again through an iterative process involving multiple readings. A codebook was subsequently developed and tested to code separately on one transcript by the two investigators. New codes were added, and the codebook revised accordingly upon consensus. The two investigators continued to code different transcripts and switched to compare the codes. This process was employed in all the transcripts and where there were fragment disagreements in the coding, an agreement was arrived at by updating the codebook. Both manual coding and Atlas.ti software (Atlas.ti version 5, Scientific Software Development GmbH, Berlin, Germany) were used for data analysis.

### Ethics considerations

Ethical approval for the study was obtained from the Kenyatta National Hospital-University of Nairobi Ethics Review Committee (KNH-UON ERC) (P151/03/2014). A research permit was also obtained from the National Commission for Science, Technology and Innovation in Kenya (#4703). Participants in the IDIs provided written informed consent, whereas FGD participants provided group, oral consent as per the ethical review approvals.

## Results

Between March and December 2015, 5 FGDs were conducted stratified by age and gender; 3 among men and 2 among women, with a total of 47 participants. The majority of FGD participants were male and married. Additionally, a total of 15 IDIs were conducted with pregnant and postpartum mothers. Interview respondents’ demographic characteristics are presented in Table 1. Several themes emerged regarding individual and community-wide influences on uptake and utilization of maternal health services, as expounded below and supported with quotations voiced by the respondents.

**Table 1:** Socio-demographic characteristics of focus group discussions and in-depth interviews participants

Characteristic	Median (IQR) or Number (Percent)	
	FGDs (***n*** = 47)	IDIs (***n*** = 15)
Age (years)	38 (27–55)	27 (24–35)
Female	15 (32)	15 (100)
Married	47 (100)	15 (100)
Education Level		
None	17 (37)	0 (0)
Primary	11 (23)	12 (80)
Secondary	19 (40)	3 (20)
Number of children	6 (3–8)	4 (2–5)

### Influence of socio-cultural norms

It emerged that while community members in this setting regularly sought services from the formal health system, socio-cultural norms continue to influence the choice of where and when to seek pregnancy, delivery and postnatal care services. This stems from the prevailing belief that ill health is as a result of evil spirits and traditional systems of health care were best-placed to deal with them. Subsequently, it has led to normalization of traditions-based health-seeking behavior.

There are some members of the community who cannot go to the hospital for health care services for whatever problem without first going to herbalists.

They say that they may go to the hospital and get injections for something whose source is from what they say are traditional practices which would make the condition worse. That is why they prefer to go for traditional treatment first even before going to the hospital.

(Female FGD participant, don’t know age, Married, 6 children, Viphalani village)

Other [people] still practice the socio-cultural beliefs of the olden days.

They say that in the olden days of our grandfathers and grandmothers, we just used to stay like that when a woman got pregnant; she would just use some roots (herbs) and she would deliver without any problem.

(Female FGD participant, 63 years, Married, 10 children, Viphalani village)

This normalization of traditions-based health-seeking behavior has led to instances where Digo women delay seeking specific maternal health services from the formal health system and has created a justification for delivering at home, only opting for a facility delivery if complications arise during the birthing process.

They [women] did not easily go to the hospital or clinics, furthermore when a woman got pregnant, she just stayed at home … and in case of any complications there was always traditional means of treatment. Certain plants were used to relieve women of abdominal pains and it has really taken long to change.

(Male FGD participant, 50 years, Married, 3 children, Magodzoni village)

They say it is good to deliver at home because there are traditional things to be done for the child, unlike in the hospital where these traditions are not performed. They believe that these practices will make the child have good health. For a child who has been born, certain practices need to be done like tying the baby with charms (*hirizi*). They believe that the child will be safe and protected without any problem if this is done and it is certain, these things will not be done at the hospital.

(Male FGD participant, 42 years, Married, 2 wives, 5 children, Kiruku village)

And that is why I told you that anybody who leaves his/her tradition is a slave.

People are used to it and that is the way the Digo tradition goes or the way they are supposed to be … so they go through that first … Before delivery, women attend clinic from month 6 or 7 of their pregnancy. But for delivery, she does it at home. Only if there are complications is when they decide to take her to hospital.

(Female FGD participant, 46 years, Married, 7 children, Kwale town)

Digo traditions are like, if my stomach is aching [labor], I will go for ‘*kufinywa nyongoo.’* If I get there, *ninachanjwa na ninawekwa mishizi* (small cuts made on body to administer powder-like herbal medicine). Then I put on my clothes and … ... I wait for my pregnancy dates and when they come, I go to deliver at hospital … but I know I will have fulfilled the traditions first.

(Female FGD participant, 63 years, Married, 10 children, Matuga village)

Respondents highlighted specific cultural practices that women undergo during pregnancy, delivery, and in the postnatal period. These include massaging of the abdomen so as to turn the baby in the womb (*kurangwa*), making incisions on the abdomen (for both mother and baby) so that herbal medicines could be applied (*kuchanjwa*) to ward off evil spirits, as well as staying in seclusion for the first forty days after delivery so as to stay away from evil eyes (*arobaini ya kwanza*).

Sometimes if the baby is not sitting properly in the womb, or is in the wrong position, you take your daughter to a *mkunga* (traditional birth attendant, TBA) for a massage so as to move the baby (*kurangwa*) … The bad effects [of doing that] are there, because as you do the massage, you don’t know exactly which part of the baby inside the womb you are pressing; you always don’t know where the baby’s head or knee is.

(Female FGD participant, 60 years, Married, 10 children, Viphalani village)

In my community, a woman has to stay indoors [for a month to 40 days] until the baby’s skin lightness disappears, that’s when you get out (local phrase used: ‘*mpaka mtoto afunike jua’).*

(Female IDI participant, 28 years, Married, 1 child, Kwale town)

I think there is a lot of influence of traditional practices especially with regards to newborn babies. You will see a baby convulsing probably due to severe pneumonia or malaria. The mother will not believe it is malaria and she will say it is “*nyun*i” (a term used by the “*Mijikenda*” to refer to convulsions associated with witchcraft-related diseases), and go for traditional medicine, but you can clearly see these are two very different things. Here the baby has fever, and there the mother saying it is “*nyuni*” and in the course of receiving the traditional healer’s services, the baby dies in the process.

(Female IDI participant, 40 years, Married, 10 children, Kizibe village)

### Role of significant matriarchal figure

Traditional birth attendants (TBAs), locally known as ‘*wakung*a’, continue to form an important community linkage for pregnancy, delivery, and postnatal care services. The TBAs are known to provide general information to pregnant mothers, facilitate home delivery and accompany them to a health facility if complications arose.

According to the Digo, we only take women to hospital if the *mkunga* has failed, that is what I always see … Nobody will take a woman to hospital at the onset of labor, and if you hear a woman has been taken there (hospital), then it is because the *mkunga* has failed … if you realize as per the woman’s condition she can deliver on her own, because most of the time she delivers at home, you don’t have to go to the hospital. You can bring the *mkunga* and she will deliver the baby.

(Male FGD participant, 43 years, Married, 2 children, Kiruku village)

In this community, women designated as *wakunga* are typically older, experienced in the practice and having had children of their own. At an individual-level, these qualities describe a significant matriarchal figure, usually the mother/grandmother, mother/grandmother-in-law or older female relatives of a pregnant woman and/or her husband. We found that in this community, these maternal figures would typically provide TBA services and play a critical role in the decision-making pathway for choice of place of delivery.

I assisted my first daughter to deliver, I also assisted my sister in-law, my granddaughter … I am not [a TBA], but I thank God …

(Female FGD participant, 46 years, Married, 7 children, Viphalani village)

Long time ago, our grandmothers, even our mothers, if a woman was in labor, the father would say we should wait first, and the mother would take charge … and that time, people never went for clinic, they did not know if the load they were carrying [pregnancy] was safe or not.

(Female IDI participant, 28 years, Married, 4 children, Mtsamviani village)

### Influence of religious norms

In this community, we found that Islamic religious norms played an integral role in women’s decision-making regarding uptake and utilization of maternal health services. Women would avoid seeking health services like delivering in a health facility if no female provider was available, in line with Islamic teachings that limit close interactions between married women and male ‘strangers’. There were also suggestions that Islam forbade use of contraception as this amounted to ‘killing’ of the unborn child.

In the Digo way of life, they are all Muslims and religion has refused (sic) us and says women must only be assisted by fellow women during child delivery.

(Male FGD participant, 51 years, Married, 8 children, Mkoyo sub-location)

Religion says it is a sin to use family planning. I follow what religion says in order not to get to hell.

(Female IDI participant, 27 years, Married, 4 children, Kizibe village)

I cannot cheat you [interviewer] because I myself did not use family planning methods. All my five children were planned by God … God also does family planning.

(Female IDI participant, 33 years, Married, 5 children, Mazumalume village)

In Islam, family planning is not allowed because it contributes to killing a living thing. It is equivalent to killing. So, most of the time in the Digo community when [women] use family planning it is because of modernization but it is not allowed by religion, because it is harmful.

(Male FGD participant, 70 years, Married, 3 wives, 7 children, Mkoyo)

I have never used any family planning method. I use the one of God only.

My stomach makes me rest once I deliver a baby, I have never gotten another pregnancy within two years. The first born is nine years, the second born now is six years, these ones (referring to the twins) are four years. Isn’t that family planning already Doctor? ...What I do is I just tell my husband if you see [menstrual] blood has come, let us wait for 11 days, then we continue with our activities [sex].

(Female IDI participant, 28 years, Married, 4 children, Matuga village)

There was an appreciation however, that as much as Islam might forbid women from being seen by other men except their husbands, this was not in line with realities in the health system. Community members were amenable to receiving health services despite the gender of the person serving them as long as they perceived them to be qualified. Regarding contraception, it was pointed out that Islam actually forbade limiting of pregnancies and not necessarily appropriate spacing of births.

Religion says that, but those who are employed there [at the hospitals] have the experience required to serve both men and women. You cannot force the government and say that you only want female employees at the hospital. So, it is true religion refuses (sic) us, but if you get to the hospital with a woman in labor, you cannot choose and insist that you want only a woman to attend to your wife. You must accept the service to be given by anybody.

(Male FGD participant, 42 years, Married, 2 wives, 5 children, Mkoyo)

Our religion does not stop a woman from planning her births, it only forbids her from stopping any more births,

(Male FGD participant, 70 years, Married, 11 children, serves as the local sheikh, Kifuku village)

### Role of gender stereotypes

Interview respondents revealed that gendered social roles among the Digo community influence access to and utilization of maternal health services. It was pointed out that the role of a woman in this community was mainly to give birth and have many children so as to preserve her husband’s lineage.

The pregnancy is yours, I′ m only waiting for the babies; my role as a husband is for the wife to inform me when there is no flour in the house and I provide, that is all.

(Male FGD participant, 70 years, Married, 11 children, serves as the local sheikh, Kifuku village)

Gender-related power imbalances were also reported with some respondents noting that Digo men, as primary providers, still retain control over decision-making. This is expressed in matters like determining the number of children to have, ultimately influencing uptake of contraception.

He wants four children, not me. He himself said he wants four. From his opinion he does not want me to use family planning.

(Female IDI participant, 23 years, Married, 1 child, Simkumbe village)

I hear my colleagues are [using] family planning [methods] but for me I have not planned. I hear they are given an injection and if they are given the injection, they stay for five years, ten years. They don’t give birth until ten years. I can use these methods but maybe I tell my husband first and he tells me it is OK to use them. I cannot decide alone.

(Female IDI participant, 24 years, Married, 2 children, Mtsamviani village)

Some men tell the women that, “If it is a matter of giving birth you have already done so. What is this business of you going to the health facility every other time? You must be having an affair with someone there.” She just sacrifices to avoid such confrontations with her husband.

(Female FGD participant, 63 years, Married, 10 children, Viphalani village)

We also found an unspoken deference to the counsel of a significant matriarchal figure regarding the number of children and use (or non-use) of contraception.

I will ask my husband first … then he will find out what his mother thinks. After that, we will do what his mother says …

(Female IDI participant, 23 years, Married, 2 children, Kwale town)

Despite these gendered cultural norms, we found that some male community members were supportive of their wives; sometimes even accompanying them to hospital to receive maternal health services.

My husband cannot accept that I deliver at home. His mother did not want but we were all (my husband and I) demanding that I go to hospital. My old man (husband) looked for a motorbike at night and came here to the hospital at night. My husband has no problem because he himself knows that hospital is of importance (sic).

(Female IDI participant, 38 years, Married, 10 children, Mazumalume village)

I have given an example of my wife who has had three deliveries through operation. The health workers had assessed her from her first delivery and advised that based on her physique, she will never deliver normally. So, I always live cautiously knowing that at any time if she is due to deliver, I have to take her to hospital in advance.

(Male FGD participant, 44 years, Married, 6 children, Ng’ombeni village)

## Discussion

The findings from this qualitative study among a remote Kenyan community highlight the influence of religious and socio-cultural norms as well as gender stereotypes on the uptake and utilization of specific maternal health services. In this setting, we found that religious and cultural norms impact the decision-making process for seeking antenatal, pregnancy and postnatal care as well as utilizing contraceptive services. Our findings show that this influence has the potential to adversely affect maternal health outcomes, especially in such settings where women have high social vulnerability secondary to gender-related power imbalances. While women in this community retain some sense of autonomy, we found that men still play a disproportionate role in the decision-making process. Importantly, our findings reveal an unspoken deference to the counsel of a prominent maternal figure, potentially related to the social role that older, experienced women in the community play during the pregnancy and birthing process.

These findings suggest that demand-creation for maternal health services in a rural, resource-limited context cannot be driven using a purely individualistic framework since health-seeking behavior is entrenched within complex religious and socio-cultural contexts. A better understanding of this context is useful in developing an efficient, inclusive framework for sustainable interventions that are culturally-acceptable and locally-responsive. For the Digo community in particular, our findings suggest that interventions seeking to improve maternal health could focus on tackling community-wide gender-stereotypes leveraging on the influence of prominent maternal figures.

While changing religious and socio-cultural norms is challenging given that they reflect deeper social structures and are reinforced by strong social institutions [17], the unspoken deference to a prominent maternal figure, is one opportunity that could be harnessed to entrench gender equitable norms among the Digo. The significant role that prominent maternal figures play in decision-making for maternal health is an important, culturally-appropriate entry point in influencing whether and how women in this setting utilize maternal health services.

Our previous studies among the Digo suggest a mixed cultural context that on the surface is paternalistic due to a strong Islamic influence. Underneath however, there’s a significant matriarchal component stemming from the community’s migratory evolution [31]. Not typical of other Bantu groupings, the migratory path of the Digo community led to close interactions with Arab/Persian traders on the East African coast. This resulted in a strong Islamic influence (predominantly patriarchal) on their culture, while still maintaining some aspects of their Bantu social stratification, kinship networks, and cultural heritage (predominantly matriarchal) [32].

The use of FGDs with community members and IDIs with pregnant and postpartum mothers allowed us to triangulate the findings and develop a better understanding of the community norms and lived experiences from the participants. We did not find diverse differences between the two methods employed to collect data, nor the different study groups of participants. Additionally, this study approach provided an opportunity to interpret community’s behavior by trying to understand the meaning they ascribe to cultural practices and symbols i.e. myths, traditions/rituals, attitudes and beliefs.

These findings need to be interpreted in the context of several limitations. First, qualitative data collection approaches depend on researcher skills and could be influenced by their personal biases. While this might lead to a subjective interpretation of data, it also allows for a more nuanced understanding of the phenomena under study [33]. In our case, data from the present study complements information from a wider overarching ethnographic study assessing the influence of cultural norms and social structure on maternal health outcomes among the Digo community. This wider study incorporates various data collection approaches including a review of information from primary and secondary sources as well as a quantitative household survey [26].

Secondly, the presence of the researcher during data collection may influence respondent’s feedback introducing social desirability bias. While this is unavoidable given the structure of the study, it could potentially lead to biased responses that misclassify study findings. In reality however, this approach reveals subtleties about the respondents and/or the topic under study that may often be missed through positivistic study approaches. In our case, while the influence of religion and cultural norms on maternal health may have been intuitive, our study approach revealed important nuances that could have been missed especially the role of prominent matriarchal figures in the community.

Finally, IDI participants in the form of pregnant and postpartum women seeking care at the local health facility were purposively selected and may not be a true reflection of the wider community. Our study aimed to provide a better understanding of the context within which maternal health interventions are implemented and how this understanding could be harnessed to improve demand for services using locally-responsive approaches. For this reason, the approach of recruiting women for interviews as they sought care was not just practical, but relevant to the study question.

## Conclusion and recommendations

We found that gender-stereotypes as well as religious and socio-cultural norms influence the uptake and utilization of maternal health services among the Digo community residing in Kwale, Kenya. This influence affected mainly the decision-making process for these services. These findings highlight the important role of demand-side factors in influencing maternal health outcomes. Maternal health programs serving similar rural and resource-limited settings could benefit from adapting interventions that address unique religious and socio-cultural influences within their target communities. This approach would harness inherent social capital and drive demand for services ensuring that programs are not only effective, but also responsive to the local context.

## Data Availability

The authors confirm that, for approved reasons, some access restrictions apply to the data underlying the findings. The authors confirm that aggregate data underlying the findings are fully available in the paper. This study was conducted with approval from the Kenyatta National Hospital - University of Nairobi Ethics and Research Committee (KNH-UON ERC), which requires that we release data from Kenyan studies (including de-identified data) only after they have provided their written approval for additional analyses. As such, data for this study will be available upon request, with written approval for the proposed analysis from the KNH-UON ERC. Their application forms and guidelines can be accessed at http://erc.uonbi.ac.ke/. If investigators wish to use the transcripts, VM and PG will assist them in contacting the KNH-UON ERC. Upon approval of the proposed secondary analysis by the KNH-UON ERC, full transcripts will be provided.

## Abbreviations

FGD: Focus Group Discussion
IDI: In-depth Interview
KDHS: Kenya Demographic and Health Survey
MNM: The Mama Na Mtoto Project
MOMI: The Missed Opportunities in Maternal and Infant Health Project
